# Immune dysregulation as a key driver of peripartum cardiomyopathy – an exploratory advanced imaging and biomarker study

**DOI:** 10.1002/ejhf.3759

**Published:** 2025-07-14

**Authors:** Julian Hoevelmann, Charle Viljoen, Tessa Kotze, Carlos Libhaber, Stephen Jermy, Morné Kahts, Fareda Jakoet‐Bassier, Petronella Samuels, Olivia Briton, Lynette Cilliers, Denise Hilfiker‐Kleiner, Peter van der Meer, Michael Böhm, Ntobeko A.B. Ntusi, Karen Sliwa

**Affiliations:** ^1^ Cape Heart Institute, Faculty of Health Sciences University of Cape Town Cape Town South Africa; ^2^ Department of Internal Medicine III ‐ Cardiology, Angiology and Intensive Care Medicine Saarland University Hospital, Saarland University Homburg Germany; ^3^ Division of Cardiology, Groote Schuur Hospital, Faculty of Health Sciences University of Cape Town Cape Town South Africa; ^4^ Cape Universities Body Imaging Centre, University of Cape Town Cape Town South Africa; ^5^ School of Physiology, Faculty of Health Sciences University of Witwatersrand Johannesburg South Africa; ^6^ Division of Biomedical Engineering, Department of Human Biology, Faculty of Health Sciences University of Cape Town Cape Town South Africa; ^7^ Department of Medicine, Groote Schuur Hospital, Faculty of Health Sciences University of Cape Town Cape Town South Africa; ^8^ Department of Dietetics Groote Schuur Hospital Cape Town South Africa; ^9^ Institute Cardiovascular Complications in Pregnancy and Oncologic Therapies Philipps University Marburg Marburg Germany; ^10^ President of the Hannover Medical School Hannover Germany; ^11^ Department of Cardiology, University Medical Center Groningen University of Groningen Groningen The Netherlands; ^12^ South African Medical Research Council Cape Town South Africa

**Keywords:** Cardiovascular magnetic resonance, Immune response, Peripartum cardiomyopathy, Positron emission tomography

## Abstract

**Aims:**

Peripartum cardiomyopathy (PPCM) is an idiopathic cardiomyopathy occurring in women in the late stages of pregnancy or in the postpartum period and is associated with significant morbidity, mortality, and persistent left ventricular dysfunction. PPCM pathogenesis involves multiple putative mechanisms including inflammation. We aimed to explore the acute inflammatory processes in PPCM using ^18^F‐fluorodeoxyglucose positron emission tomography‐computed tomography (^18^FDG‐PET‐CT), cardiovascular magnetic resonance (CMR), and inflammasome profiling.

**Methods and results:**

Women with a new diagnosis of PPCM (*n* = 10, all within 3 months postpartum), five healthy postpartum controls (HPC), and five healthy non‐postpartum controls (HNPC), underwent ^18^FDG‐PET‐CT, CMR, and serum inflammatory proteomic profiling. PPCM patients had a median age of 34 years (interquartile range [IQR] 30.3–38.5, similar in control groups), and a median parity of 3 (IQR 1–4). PPCM patients presented with severe, symptomatic heart failure (all New York Heart Association functional class III/IV), reduced median left ventricular ejection fraction of 35.5% (IQR 18.1–37.9). PPCM and HPC groups showed higher myocardial and splenic ^18^FDG uptake compared to HNPC. On CMR, myocardial interstitial fibrosis (elevated T1 time, extracellular volume, and late gadolinium enhancement mass) was solely present in PPCM. Inflammatory profiling showed pro‐inflammatory cytokine dysregulation in PPCM (elevated neutrophil‐to‐lymphocyte ratio, C‐reactive protein, interleukin‐6, tumour necrosis factor‐α, chemokine (C‐C motif) ligand 3, hepatocyte growth factor, chemokine (C‐X‐C motif) ligand 10 and colony‐stimulating factor‐1) compared to controls.

**Conclusions:**

Patients with PPCM exhibited a dysregulated immune response, associated with early myocardial interstitial fibrosis and adverse cardiac remodelling. This highlights the importance of rapid initiation of guideline‐directed medical therapy especially with drugs documented to have anti‐fibrotic effects.

## Introduction

Peripartum cardiomyopathy (PPCM) is a pregnancy‐associated form of heart failure (HF) with acute onset affecting women without known cardiomyopathies or other forms of cardiovascular disease. PPCM is linked to significant premature mortality, arrhythmias, and can result in irreversible cardiac remodelling in a substantial proportion of patients.^1^, ^2^ It occurs globally, however, with marked regional differences in epidemiology and outcomes.^3^, ^4^ Extensive research elucidated elements of the pathophysiology of PPCM in murine models and humans. Contemporary hypotheses suggest a multi‐hit model for the development of PPCM, whereby susceptible women (i.e. those with genetic predisposition, at extremes of age, with multiple pregnancies and/or nutritional deficiencies) experience unbalanced oxidative stress that promotes an angiogenic and metabolic imbalance and subsequently leads to HF in the peripartum period.^2^, ^5^, ^6^, ^7^, ^8^ Among those, prolactin plays the most important role, as oxidative stress triggers cathepsin D to cleave the full‐length nursing hormone prolactin into an anti‐angiogenic 16‐kDa fragment.^9^, ^10^ The understanding of the role of prolactin led to the utilization of disease‐specific therapeutic prolactin blockers, bromocriptine/cabergoline, which improve outcomes.^11^, ^12^, ^13^ Aside from bromocriptine, anti‐inflammatory therapies have been beneficial in an African PPCM population.^14^

However, the causes of oxidative stress and the ensuing inflammatory processes remain poorly delineated. The aim of this study was to explore the inflammatory processes directly activated in the acute phase of a *de novo* HF such as PPCM using whole‐body and cardiac ^18^F‐fluorodeoxyglucose positron emission tomography‐computed tomography (^18^FDG‐PET‐CT), cardiovascular magnetic resonance (CMR), and serum inflammatory proteomic profiling and to delineate whether the inflammatory processes are limited to the heart, or also involve extra‐cardiac or systemic inflammation.

## Methods

### Study participants

**Figure 1:**
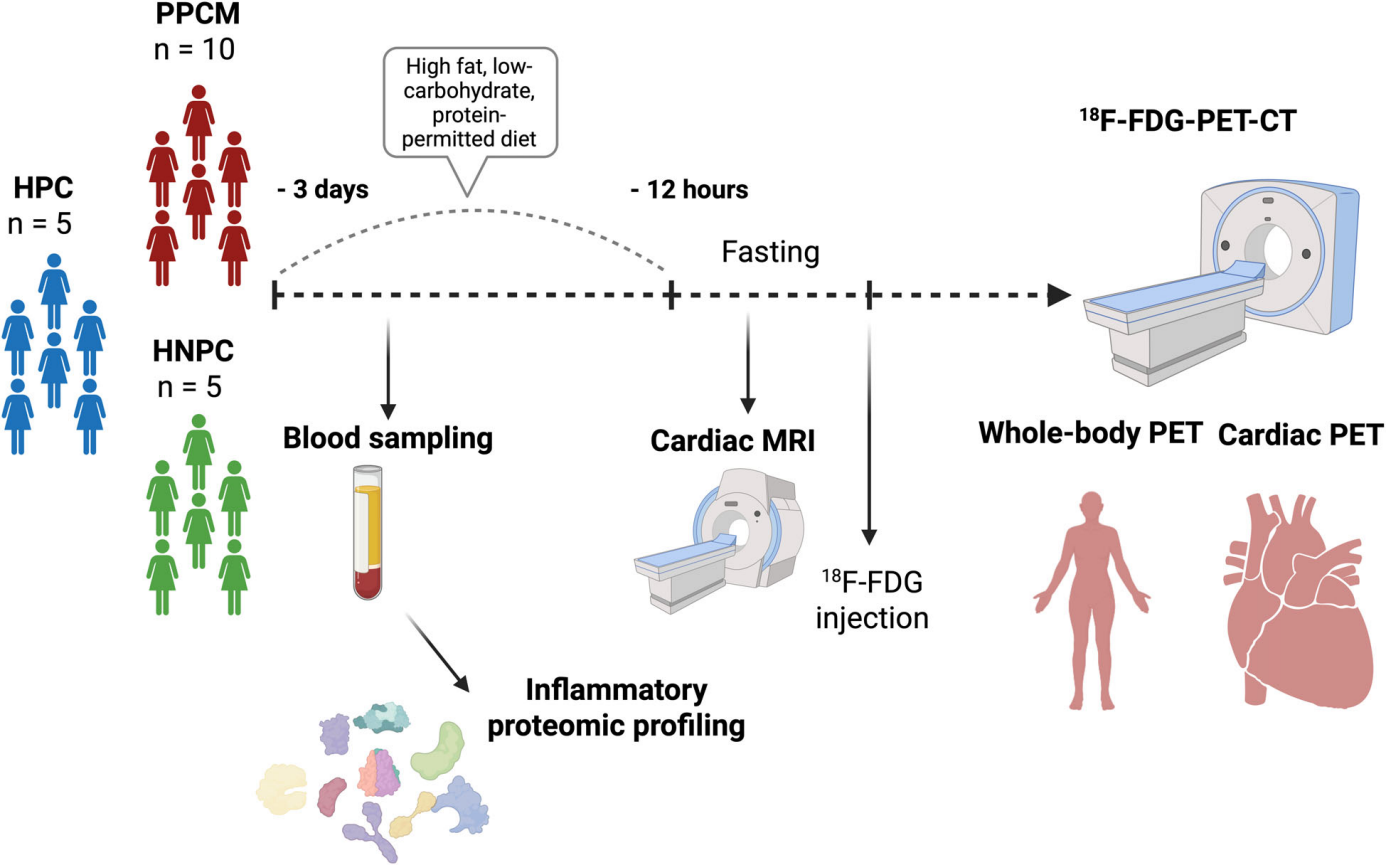
Study flow. ^18^F‐FDG, ^18^F‐fluorodeoxyglucose; HNPC, healthy non‐postpartum controls; HPC, healthy postpartum controls; MRI, magnetic resonance imaging; PET, positron emission tomography; PPCM, peripartum cardiomyopathy.

Ethical approval from the University of Cape Town (UCT) Faculty of Health Sciences (FHS) Human Research Ethics Committee (HREC) (UCT FHS HREC reference number 209/2020) and institutional permission (RC 307/WC_202111_039) was obtained. The study was performed in accordance with the Declaration of Helsinki and all participants provided written informed consent prior to study inclusion. All participants received detailed counselling about the benefits and risks of the study participation. Consecutive consenting patients with acute PPCM (*n* = 11) had to be within the first three postpartum months and without diabetes mellitus or any infectious diseases, such as HIV or tuberculosis. Detailed eligibility criteria are provided in online supplementary *Table Appendix* *S1*. One participant with PPCM had to be excluded due to non‐adherence to the dietary preparation protocol prior to the ^18^FDG‐PET‐CT scan, resulting in a final analysis involving 10 PPCM participants. In addition, we recruited five healthy postpartum controls (HPC, i.e. within the first 6 months postpartum) as well as five healthy non‐postpartum controls (HNPC, i.e. no pregnancy in the 24 months prior to enrolment to the study), all aged ≥30 years. The age cut‐off for the control groups was based on a publication of the Netherlands Commission on Radiation Dosimetry, ‘Human Exposure to Ionizing Radiation for Clinical and Research Purposes: Radiation Dose and Risk Estimates’, in order to balance comparability of the study groups in terms of age and reducing the long‐term risks of ionizing radiation exposure to healthy participants. Data collection included sociodemographic parameters, medical and obstetric history, clinical assessment, including New York Heart Association functional class (NYHA FC), routine laboratory investigations, 12‐lead electrocardiogram (ECG), echocardiogram, CMR, as well as cardiac and whole‐body ^18^FDG‐PET‐CT scans. Bloods were collected for inflammatory biomarker measurements on the day of the study recruitment. A visual description of the study flow is presented in *Figure* *1*.

### Dietary preparation for cardiac ^18^F‐FDG‐PET‐CT

All participants were instructed to follow a standardized high‐fat, high‐protein, very low‐carbohydrate diet for 3 days prior to the ^18^FDG‐PET‐CT scan to suppress glucose utilization of the myocardium. The dietary protocol was developed in conjunction with a specialist dietician and individually adjusted to the participants' daily recommended calory intake based on ideal body weight. A range of commercialized available nutritional formulations were utilized to simplify implementation, improve adherence, and infer standardization. The enteral nutrition supplements included interchangeable options to accommodate patient palate preferences and allow for procurement flexibility. Utilizing standardized enteral nutritional products ensured controlled intake of macronutrients (with an emphasis of total caloric intake in the context of controlled fat and carbohydrate ratios) and minimized risk of accidental non‐adherence to dietary restrictions. Participants were asked to fast for a minimum 12 h before the scan, during which time they could only drink plain water. None of the patients with PPCM and the postpartum patients were breastfeeding at the time of study inclusion.

### ^18^FDG‐PET‐CT

^18^FDG PET‐CT scans were performed on a Biograph mCT Flow 64 PET‐CT scanner (Siemens Healthineers, Erlangen, Germany) at the Cape Universities Body Imaging Centre (CUBIC). First, a low‐dose CT scan cardiac acquisition (120 kVp, 11 mAs, pitch 1, 3 mm) and whole body (120 kVp, 30 mAs, pitch 1, 5 mm) based on participants' mass was acquired for attenuation correction of PET images. ^18^FDG‐PET doses were calculated 2.8 x per patient weight; cardiac scan at 45 min, 10 min per bed position for all participants; whole body scan at 60 min with continuous bed motion for all participants.

All ^18^FDG PET‐CT scans were reviewed by two nuclear medicine specialists (TK and CL) with >10‐year experience in ^18^FDG‐PET‐CT reporting. The nuclear physicians were blinded to which cohort the participants belonged to. The images were reported independently and discrepancies in reported results were resolved by consensus. Myocardial and extra‐cardiac ^18^FDG activity was assessed by target‐to‐background ratio (TBR). This value was derived by calculating a volume of interest' (VOI) maximum standardized uptake divided by the VOI of the blood pool mean' standardized uptake value. If increased cardiac uptake was found, it was described as focal or diffuse, and its location within the heart was depicted. The maximum standardized uptake value (SUVmax) for each thoracic vertebra was calculated using a VOI, and overall bone marrow activity was derived as the mean of SUVmax of all thoracic vertebrae. The SUVmax for the liver, spleen, breast, and uterus was derived by placing a VOI adjusted to the organ size.

### Cardiovascular magnetic resonance protocol

Participants were scanned on a whole‐body magnetic resonance imaging system (3 T Skyra, Siemens Healthineers, Erlangen, Germany), using an 18‐channel body array coil, integrated spine coil and standardized CMR protocol. Images were ECG‐gated and acquired during arrested expiration breath‐hold. The standardized CMR protocol included standard long‐axis (four‐, two‐, three‐chamber) and consecutive short‐axis cine images covering the left ventricle (LV) from the base to the apex. Cine images were used to perform left and and right ventricular chamber assessment including ventricular and valvular function, mass, and chamber sizes and global strain values were calculated using tissue tracking. Late gadolinium enhancement (LGE) images were acquired 6–10 min after intravenous administration of 0.2 mmol/kg body weight Magnevist (Bayer HealthCare, Leverkusen, Germany). LGE images were based on a T1‐weighted phase‐sensitive recovery sequence. The inversion time (TI) was individually adjusted for optimal nulling of normal myocardium. LGE images were assessed for presence of fibrosis and LGE mass was determined using semiautomatic quantitative LGE analysis using a cut‐off of three standard deviations above the mean signal intensity of a chosen non‐enhanced region of the myocardium. Quantitative LGE analysis was performed on all subjects regardless of presence of fibrosis. Native T1 and T2 mapping were performed on three parallel short‐axis slices (LV basal, mid‐ventricular, apical) and the horizontal long‐axis view. Native T1 mapping was performed using the Modified Look‐Locker Inversion Recovery (MOLLI) sequence with a 5(3)3 scheme.^15^ Post‐contrast T1 mapping was done 15 min after contrast injection using MOLLI with a 4(1)3(1)2 scheme. The T1 value of the blood pool was determined by manually drawing a region of interest in the LV cavity taking care to exclude papillary muscles and trabeculae. Native and post‐contrast myocardial and blood pool T1 values were then used to calculate the extracellular volume fraction (ECV).^15^ Myocardial T2 mapping was performed using a T2‐prepared fast low‐angle shot (FLASH) sequence. CMR post‐processing was performed by two independent readers with over 5‐year experience (SJ and MK) using CVI (v5.17, Circle Cardiovascular, Calgary, Canada) following standardized guidelines.^16^

### Biomarker measurements

Ethylenediamine tetraacetic acid (EDTA) blood was drawn at the time of PPCM diagnosis for participants with PPCM, and at the time of study inclusion for HPC and HNPC. Blood was centrifuged immediately, and the supernatant was separated. The plasma samples were stored at −80°C before being analysed at the Centre for Proteomic and Genomic Research (CPGR) at UCT. The Olink® (Uppsala, Sweden) Target 48 Cytokine panel was measured for all participants according to manufacturer instructions. A complete list of the inflammatory biomarkers measured is provided in online supplementary *Table* *S2*.

### Statistical analysis

Data were collected on Research Electronic Data Capture (REDCap Version 9.5.36), a secure electronic database hosted by UCT, before being exported to Stata (Version 17, Stata Corp., College Station, TX, USA) for statistical analysis. Descriptive statistics were used to summarize data. Continuous variables were expressed by their means with standard deviations for parametric data or median with interquartile range (IQR) for non‐parametric data, and compared by using Student's *t*‐test, Wilcoxon rank‐sum test or Mann–Whitney U test as appropriate. Categorical variables were expressed by their frequency and percentage and were compared with *χ* ^2^ and Fisher's exact tests depending on the sample size. For biomarker measurements, comparisons between three groups were performed using ANOVA. *P*‐values from this test were adjusted for multiple testing using the Benjamini–Hochberg method. Assays that had an adjusted global *F*‐test *p*‐value < 0.05 are then moved on to a post‐hoc analysis to compare PPCM versus HPC, PPCM versus HNPC, and HPC versus HNPC to determine which specific groups differ from each other. These *p*‐values were then adjusted using the Tukey method and means were estimated using the Emmeans package in the statistical software R. Spearman *r* was reported as correlation coefficient for non‐normally distributed variables. A *p* < 0.05 was considered to indicate statistical significance.

## Results

### Clinical characteristics

Overall, participants had a median age of 34 years (IQR 30.3–38.5, with no significant differences between groups) and a median parity of 3 (IQR 2–3.5) at the time of study enrolment. As shown in *Table* *1*, all participants with PPCM were symptomatic of HF at the time of recruitment (NYHA FC III in 60% and NYHA FC IV in 40%). The median time between delivery and presentation was 16 days (IQR 7–65) for participants with PPCM. The HPC were recruited after a median time of 97 days (IQR 50–106) after their delivery (*p* = 0.12). The median heart rate was higher in women with PPCM (99.5 bpm [IQR 92–108]) as compared to HPC (63 bpm [IQR 57–68]), and HNPC (62 bpm [IQR 56–67], *p* = 0.024). The corrected QT interval by Bazett formula (QTcB) was significantly longer in participants with PPCM (489.5 ms [IQR 468–505]) compared to HPC (416 ms [IQR 394–422]) and HNPC (396 ms [IQR 393–417], *p* = 0.006).

**Table 1:** Baseline clinical characteristics of patients with peripartum cardiomyopathy, healthy postpartum controls and healthy non‐postpartum controls

	Total (*n* = 20)	PPCM (*n* = 10)	HPC (*n* = 5)	HNPC (*n* = 5)	*p*‐value
Age at presentation (years)	34.0 (30.3–38.5)	32.8 (22.7–37.3)	33.1 (32.7–37.7)	38.8 (31.1–39.5)	0.33
Ethnicity					1.00
African or Black	12 (60.0)	6 (60.0)	3 (60.0)	3 (60.0)	
Coloured or mixed	8 (40.0)	4 (40.0)	2 (40.0)	2 (40.0)	
Time between delivery and presentation (days)	38.0 (9.0–104.0)	16.0 (7.0–65.0)	97.0 (50.0–106.0)		0.12
Parity	3.0 (2.0–3.5)	3.0 (1.0–4.0)	3.0 (2.0–4.0)	2.0 (2.0–2.0)	0.32
BMI (kg/m^2^)	25.4 (24.2–32.3)	24.4 (23.4–32.3)	30.7 (25.4–33.1)	25.0 (24.2–27.2)	0.26
NYHA FC at presentation					<0.001
I	10 (50.0)	0 (0.0)	5 (100.0)	5 (100.0)	
III	6 (30.0)	6 (60.0)	0 (0.0)	0 (0.0)	
IV	4 (20.0)	4 (40.0)	0 (0.0)	0 (0.0)	
Systolic BP (mmHg)	121.5 (115.5–131.5)	120.0 (118.0–130.0)	124.0 (121.0–126.0)	116.0 (115.0–134.0)	0.97
Diastolic BP (mmHg)	79.0 (68.0–85.5)	76.5 (67.0–89.0)	76.0 (64.0–80.0)	81.0 (78.0–84.0)	0.61
QRS rate (bpm)	70.0 (59.5–99.5)	99.5 (92.0–108.0)	63.0 (57.0–68.0)	62.0 (56.0–67.0)	0.024
Rhythm					0.13
Sinus rhythm	10 (50.0)	4 (40.0)	3 (60.0)	3 (60.0)	
Sinus tachycardia	5 (25.0)	1 (10.0)	2 (40.0)	2 (40.0)	
Sinus bradycardia	5 (25.0)	5 (50.0)	0 (0.0)	0 (0.0)	
QRS width (ms)	80.0 (72.0–84.0)	81.0 (76.0–84.0)	80.0 (80.0–88.0)	72.0 (69.0–72.0)	0.14
T‐wave inversion	8 (40.0)	8 (80.0)	0 (0.0)	0 (0.0)	0.001
QTc interval by Bazett (ms)	446.7 (395.3–489.5)	489.5 (468.3–505.2)	416.0 (394.4–421.6)	396.3 (393.2–416.8)	0.006
Leucocytes (10^3^/μl)	8.2 (5.8–11.0)	9.6 (8.1–14.0)	5.8 (5.8–8.4)	5.8 (5.6–6.3)	0.023
Neutrophils (%)	57.0 (51.0–66.9)	68.3 (61.7–72.2)	52.2 (52.0–54.0)	49.9 (47.7–54.1)	0.023
Lymphocytes (%)	34.0 (22.3–40.5)	22.3 (17.3–26.4)	39.0 (38.9–40.0)	41.5 (39.9–43.4)	0.021
NLR	1.7 (1.3–3.1)	3.1 (2.3–4.2)	1.3 (1.3–1.3)	1.1 (1.1–1.4)	0.014
Monocytes (%)	6.4 (5.2–7.9)	8.4 (6.8–9.8)	6.0 (5.1–6.4)	6.3 (5.4–7.7)	0.15
Eosinophils (%)	1.6 (0.1–3.0)	0.4 (0.1–0.9)	3.0 (2.1–3.6)	2.1 (0.5–2.5)	0.086
Basophils (%)	0.3 (0.1–0.4)	0.4 (0.3–0.6)	0.2 (0.1–0.4)	0.1 (0.0–0.4)	0.37
Immature cells (%)	0.2 (0.0–0.5)	0.7 (0.5–0.8)	0.2 (0.0–0.2)	0.0 (0.0–0.0)	0.022
Haemoglobin (g/dl)	11.2 (9.7–12.4)	10.2 (9.2–10.9)	11.7 (11.6–12.5)	12.2 (11.7–13.5)	0.17
Haematocrit (%)	40.0 (30.0–40.0)	30.0 (30.0–40.0)	40.0 (30.0–40.0)	40.0 (40.0–40.0)	0.32
MCV (fl)	89.4 (80.6–91.5)	88.9 (77.7–92.4)	90.0 (89.0–90.0)	90.0 (71.0–91.0)	0.69
MCH (pg)	27.9 (25.8–29.5)	26.8 (25.2–28.7)	29.0 (29.0–30.0)	29.0 (20.0–29.0)	0.22
Red cell distribution width	14.4 (13.2–18.5)	17.4 (14.2–19.9)	12.9 (12.1–13.8)	14.0 (12.5–16.4)	0.040
Platelets (/μl)	337.0 (288.0–406.0)	316.5 (283.0–490.0)	364.0 (359.0–387.0)	310.0 (293.0–380.0)	0.60
Potassium (mmol/L)	4.1 (3.9–4.4)	3.9 (3.9–4.4)	4.7 (4.4–5.1)	4.0 (3.9–4.1)	0.039
Creatinine (μmol/L)	64.5 (54.0–79.5)	79.5 (59.0–87.0)	57.0 (54.0–64.0)	57.0 (54.0–67.0)	0.12
Fe (μmol/L)	8.6 (5.0–16.3)	5.3 (3.6–8.4)	9.9 (6.0–13.4)	22.5 (16.3–26.2)	0.004
Transferrin (mg/dl)	2.7 (2.4–3.2)	2.4 (2.3–3.2)	2.6 (2.4–2.7)	3.3 (3.0–22.0)	0.10
TSAT (%)	13.0 (7.0–22.0)	8.0 (7.0–11.0)	14.0 (9.0–22.0)	22.0 (22.0–31.0)	0.021
Ferritin (μg/L)	46.0 (27.5–79.5)	73.0 (51.0–88.0)	45.0 (34.0–45.0)	20.0 (12.0–32.0)	0.029
NT‐proBNP (pg/ml)	518.0 (43.0–4781.0)	4781.0 (1655.0–7459.0)	53.0 (23.0–112.5)	43.0 (23.0–49.0)	0.002
CRP (mg/L)	3.7 (1.9–37.0)	40.5 (10.5–76.5)	3.4 (2.0–9.0)	1.0 (1.0–1.9)	0.003
Loop diuretics	9 (45.0)	9 (90.0)	0 (0.0)	0 (0.0)	<0.001
MRA	7 (35.0)	7 (70.0)	0 (0.0)	0 (0.0)	0.005
ACE‐I or ARB	10 (50.0)	10 (100.0)	0 (0.0)	0 (0.0)	<0.001
Beta‐blocker	10 (50.0)	10 (100.0)	0 (0.0)	0 (0.0)	<0.001
Bromocriptine	6 (30.0)	6 (60.0)	0 (0.0)	0 (0.0)	0.014

Data presented as *n* (%), or median (interquartile range).

ACE‐I, angiotensin‐converting enzyme inhibitor; ARB, angiotensin receptor blocker; BMI, body mass index; BP, blood pressure; CRP, C‐reactive protein; Fe, serum iron; HNPC, healthy non‐postpartum controls; HPC, healthy postpartum controls; MCH, mean corpuscular haemoglobin; MCV, mean corpuscular volume; MRA, mineralocorticoid receptor antagonist; NLR, neutrophil‐to‐lymphocyte ratio; NT‐proBNP, N‐terminal pro‐B‐type natriuretic peptide; NYHA FC, New York Heart Association functional class; PPCM, peripartum cardiomyopathy; QTc, corrected QT interval; RDW, red cell distribution width; TSAT, transferrin saturation.

### Inflammatory proteomic profiling

Routine laboratory analysis showed that leucocyte counts were highest in participants with PPCM (9.6 10^3^/μl [IQR 8.1–14.0]) compared to HPC (5.8 10^3^/μl [IQR 5.8–8.4]) and HNPC (5.8 10^3^/μl [IQR 5.6–6.3], *p* = 0.023). Similarly, C‐reactive protein (CRP) was significantly elevated in participants with PPCM (40.5 mg/L [IQR 10.5–76.5]) as compared to HPC (3.4 mg/L [IQR 2.0–9.0]) and HNPC (1.0 mg/L [IQR 1.0–1.9], *p* = 0.003). On differential blood count, patients with PPCM had the highest proportion of neutrophils and lowest proportion of lymphocytes as compared to HPC and HNPC. Neutrophil‐to‐lymphocyte ratio (NLR) was highest in patients with PPCM (3.1) versus HPC (1.3) and HNPC (1.1). Biochemistry also showed that patients with PPCM had iron deficiency (Fe 5.3 μmol/L [IQR 3.6–8.4], transferrin 2.4 mg/dl [IQR 2.3–3.2], transferrin saturation 8% [IQR 7.0–11.0] and ferritin 73.0 μg/L [IQR 51.0–88.0]). The median NT‐proBNP was markedly up‐regulated in patients with PPCM (4781 pg/ml [IQR 1655.0–7459.0]) in keeping with acute HF and compared to 53 pg/ml [IQR 23.0–112.5] in HPC and 43.0 pg/ml [23.0–49.0] in HNPC, respectively.

**Figure 2:**
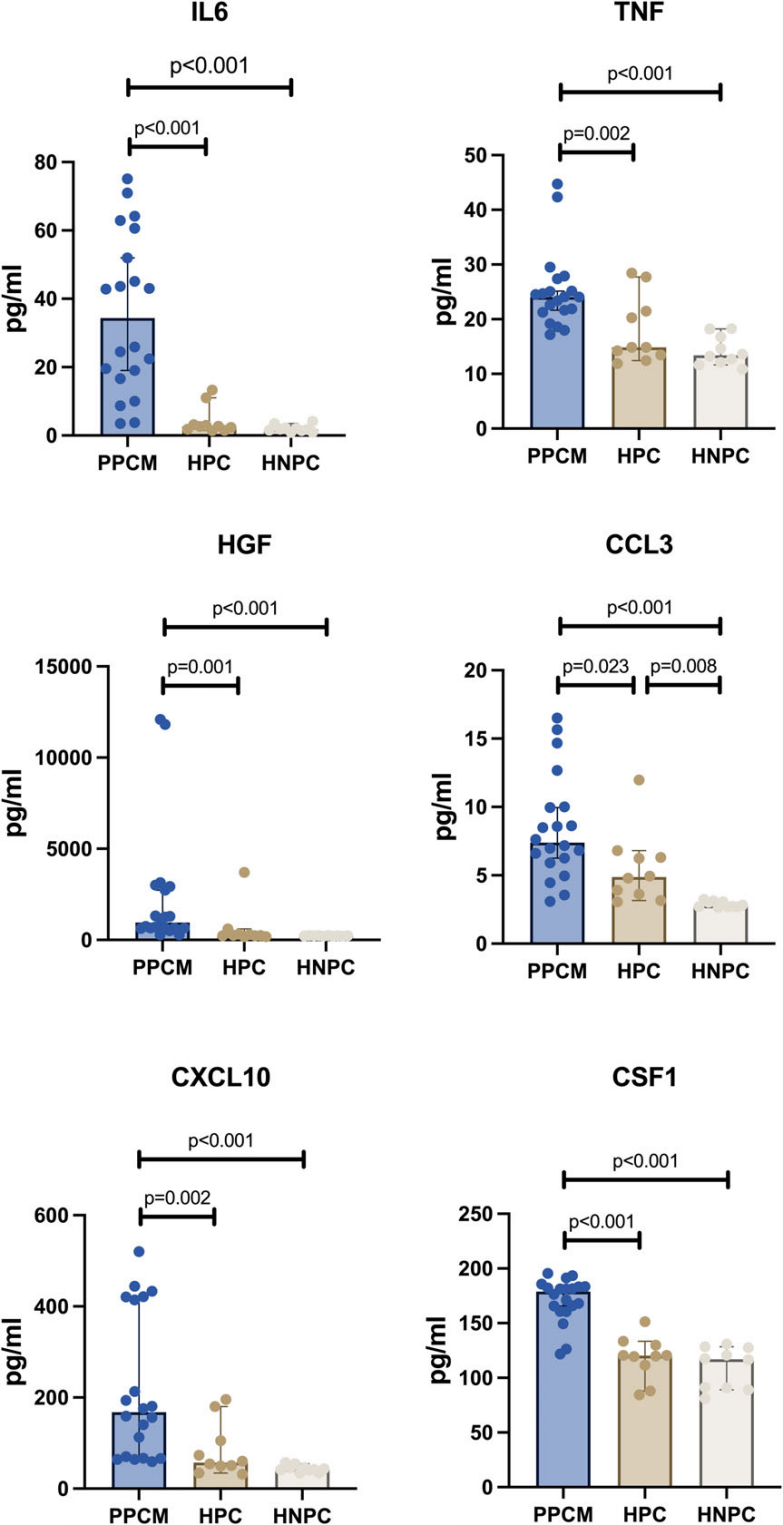
Inflammatory biomarkers levels as compared between peripartum cardiomyopathy (PPCM) patients, healthy postpartum controls (HPC) and healthy non‐postpartum controls (HNPC). CCL3, chemokine (C‐C motif) ligand 3; CSF1, colony stimulating factor‐1; CXCL10, chemokine (C‐X‐C motif) ligand 10; HGF, hepatocyte growth factor; IL6, interleukin‐6; TNF, tumor necrosis factor.

Inflammasome profiling revealed that among the 45 cytokines assessed, 23 exhibited notable differences between groups (online supplementary *Table* *S3*). Further categorization by sub‐group analysis revealed that circulatory interleukin (IL)‐6, macrophage colony‐stimulating factor‐1 (CSF1), chemokine (C‐X‐C motif) ligand 10 (CXCL10), tumour necrosis factor (TNF), hepatocyte growth factor (HGF) and chemokine (C‐C motif) ligand 3 (CCL3) were significantly upregulated in PPCM, when compared to HPC and HNPC (*Figure* *2*, online supplementary *Table* *S4*). In addition, CCL3 was also significantly elevated in both PPCM and HPC as compared to HNPC. In contrast, chemokine (C‐C motif) ligand 8 (CCL8), IL‐4 and IL‐13 were significantly down‐regulated in PPCM compared to HPC and lymphotoxin‐alpha was significantly down‐regulated in PPCM compared to HNPC (online supplementary *Table* *S4*).

### Cardiovascular magnetic resonance

**Figure 3:**
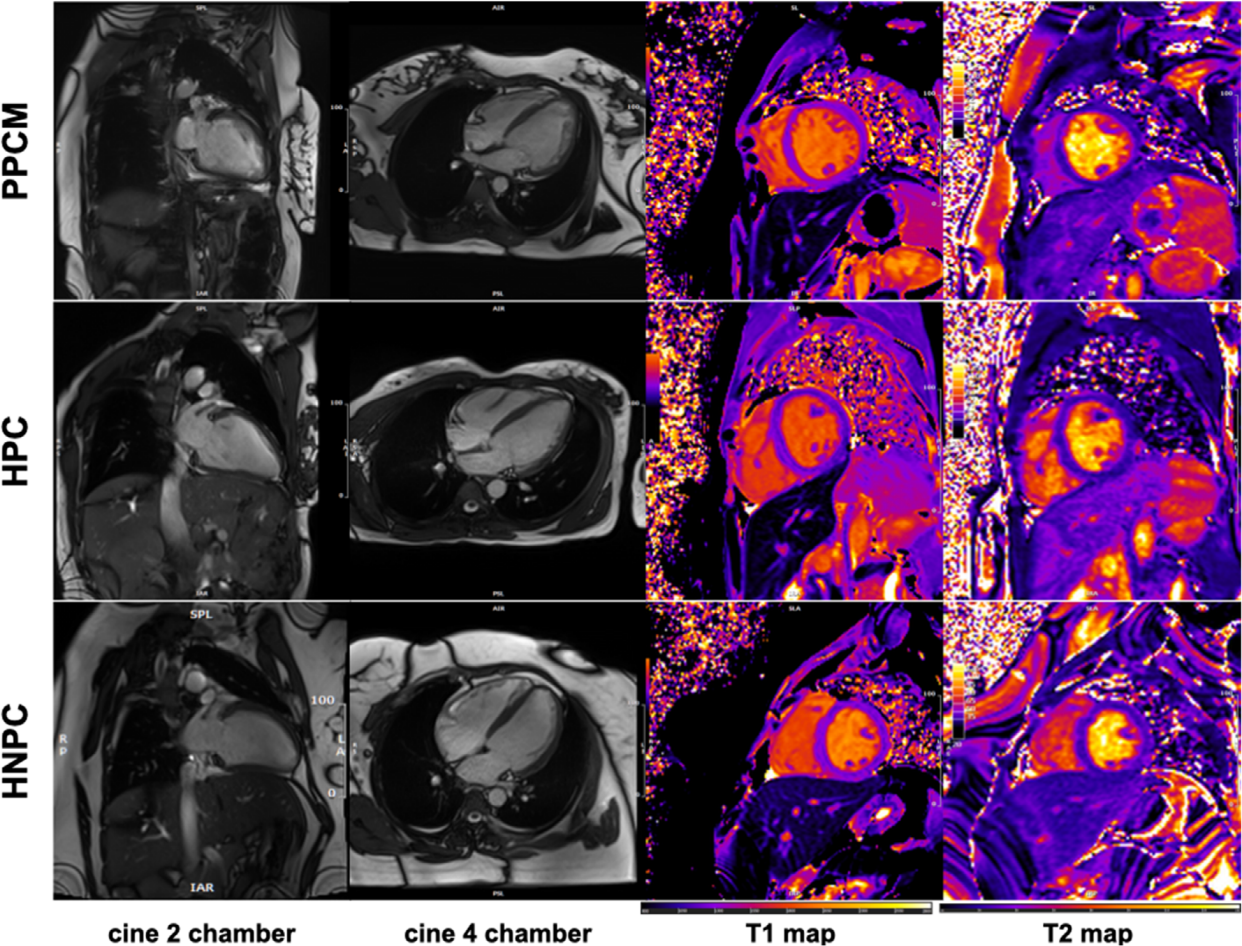
Representative cardiovascular magnetic resonance images of peripartum cardiomyopathy (PPCM) patients, healthy postpartum controls (HPC) and healthy non‐postpartum controls (HNPC) displaying a cine two chamber (first row), cine four chamber (second row) as well as short axes at papillary muscle level of native T1 map (third row) and T2 map (fourth row).

Patients with PPCM exhibited significantly higher LV end‐diastolic and end‐systolic volumes indexed to body surface area (*Table* *2*). Indexed LV mass and LV end‐diastolic diameter were significantly higher in PPCM patients compared to controls (*p* < 0.001, *p* = 0.004, respectively). In contrast, left and right atrial areas showed no significant differences between participants with PPCM and controls. Both left and right ventricular systolic function as well global longitudinal, circumferential, and radial strain were significantly impaired in PPCM compared to the control groups. An illustration of representative CMR images is provided in *Figure* *3*.

**Table 2:** Cardiovascular magnetic resonance of patients with peripartum cardiomyopathy, healthy postpartum controls and healthy non‐postpartum controls

	All (*n* = 20)	PPCM (*n* = 10)	HPC (*n* = 5)	HNPC (*n* = 5)	*p*‐value
LVEDD (mm)	51.8 (49.6–60.1)	60.0 (54.2–61.2)	50.9 (48.3–51.7)	48.2 (46.3–50.1)	0.004
LVEF (%)	40.2 (35.5–63.8)	35.5 (18.1–37.9)	63.6 (62.0–64.1)	64.8 (59.2–67.2)	<0.001
LVEDV (ml)	172.1 (162.1–226.1)	226.1 (211.6–242.6)	167.3 (164.8–176.7)	130.6 (124.4–137.6)	0.004
LVESV (ml)	98.0 (59.0–147.8)	147.8 (131.8–184.2)	63.6 (63.5–64.8)	44.6 (43.3–54.6)	<0.001
LVSV (ml)	81.3 (65.3–96.1)	65.3 (40.7–83.0)	109.0 (103.7–113.2)	87.8 (79.7–93.0)	0.012
LVM (g)	99.4 (71.2–118.1)	118.1 (110.0–125.6)	87.8 (82.9–94.0)	61.3 (60.1–70.3)	<0.001
LVEDVi (ml/m^2^)	106.1 (82.1–124.8)	124.8 (114.6–136.6)	92.0 (79.8–104.3)	79.2 (70.7–84.4)	0.001
LVESVi (ml/m^2^)	63.6 (29.4–81.5)	81.5 (72.9–106.1)	33.1 (28.8–39.3)	27.4 (26.0–30.0)	<0.001
LVSVi (ml/m^2^)	45.1 (38.8–53.6)	39.9 (25.9–44.5)	54.0 (46.9–59.0)	53.2 (45.8–56.4)	0.017
LVMi (g/m^2^)	54.2 (40.5–64.5)	64.5 (61.1–75.5)	45.6 (42.2–45.7)	37.2 (35.8–39.7)	<0.001
RVEF (%)	56.0 (43.9–61.2)	43.9 (21.6–52.5)	61.1 (57.5–62.4)	61.3 (57.5–64.5)	0.002
RVEDV (ml)	152.4 (129.3–193.9)	177.0 (146.8–224.1)	170.0 (155.9–172.6)	132.2 (126.4–143.9)	0.35
RVESV (ml)	67.8 (54.5–109.1)	106.6 (67.6–163.1)	66.1 (55.7–68.0)	54.7 (46.9–55.6)	0.12
RVEDVi (ml/m^2^)	79.7 (73.2–111.3)	96.1 (73.8–134.2)	78.1 (77.2–88.5)	80.1 (72.6–88.3)	0.48
RVESVi (ml/m^2^)	35.5 (29.5–64.1)	61.2 (36.6–91.1)	33.2 (28.0–34.4)	30.1 (28.4–34.1)	0.037
LA area (cm^2^)	21.9 (19.3–24.0)	22.8 (21.1–24.0)	23.2 (20.1–24.2)	19.5 (18.9–20.2)	0.16
RA area (cm^2^)	19.8 (16.4–25.2)	21.6 (16.4–25.9)	19.1 (18.5–22.7)	18.7 (16.0–21.0)	0.62
Global circumferential strain (%)	−13.4 (−20.3 to −9.5)	−10.0 (−12.5 to −5.8)	−20.3 (−21.6 to −19.1)	−20.2 (−22.6 to −18.4)	0.002
Global longitudinal strain (%)	−12.2 (−19.6 to −8.8)	−9.6 (−11.8 to −6.2)	−19.7 (−20.0 to −19.6)	−19.3 (−20.0 to −18.3)	0.001
Global radial strain (%)	18.9 (12.6–39.0)	13.5 (6.8–17.3)	36.5 (32.5–39.5)	39.2 (33.9–44.7)	0.001
Native T1 (ms)	1291.8 (1249.2–1377.8)	1377.8 (1311.3–1409.7)	1249.3 (1249.0–1254.7)	1226.2 (1212.3–1260.3)	0.002
ECV (%)	32.1 (28.4–36.3)	36.3 (32.1–37.0)	28.9 (28.6–30.5)	28.0 (26.3–32.1)	0.028
T2 (ms)	41.0 (39.3–42.2)	41.0 (39.3–42.3)	41.3 (40.7–41.7)	39.2 (38.6–42.0)	0.67
LGE mass (g)	7.7 (3.9–18.6)	18.6 (15.7–20.7)	2.9 (2.8–3.7)	7.3 (7.1–7.7)	0.001
Fibrosis (%)	7 (36.8)	7 (77.8)	0 (0.0)	0 (0.0)	0.002
Mitral regurgitation (%)					0.012
None	11 (55.0)	1 (10.0)	5 (100.0)	5 (100.0)	
Mild	2 (10.0)	2 (20.0)	0 (0.0)	0 (0.0)	
Moderate	3 (15.0)	3 (30.0)	0 (0.0)	0 (0.0)	
Severe	4 (20.0)	4 (40.0)	0 (0.0)	0 (0.0)	
Tricuspid regurgitation (%)					0.029
None	13 (65.0)	3 (30.0)	5 (100.0)	5 (100.0)	
Mild	4 (20.0)	4 (40.0)	0 (0.0)	0 (0.0)	
Severe	3 (15.0)	3 (30.0)	0 (0.0)	0 (0.0)	
Pericardial effusion (%)	8 (40.0)	8 (80.0)	0 (0.0)	0 (0.0)	0.001

ECV, extracellular volume fraction; HNPC, healthy non‐postpartum controls; HPC, healthy postpartum controls; LA, left atrial; LGE, late gadolinium enhancement; LVEDD, left ventricular end‐diastolic diameter; LVEDV, left ventricular end‐diastolic volume; LVEDVi, left ventricular end‐diastolic volume index; LVEF, left ventricular ejection fraction; LVESD, left ventricular end‐systolic diameter; LVESV, left ventricular end‐systolic volume; LVESVi, left ventricular end‐systolic volume indexed; LVM, left ventricular mass; LVMi, left ventricular mass indexed; LVSV, left ventricular stroke volume; LVSVi, left ventricular stroke volume indexed; PPCM, peripartum cardiomyopathy; RA, right atrial; RVEF, right ventricular ejection fraction; RVESV, right ventricular end‐systolic volume; RVESVi, right ventricular end‐systolic volume indexed; RVSV, right ventricular stroke volume; RVSVi, right ventricular stroke volume indexed.

**Figure 4:**
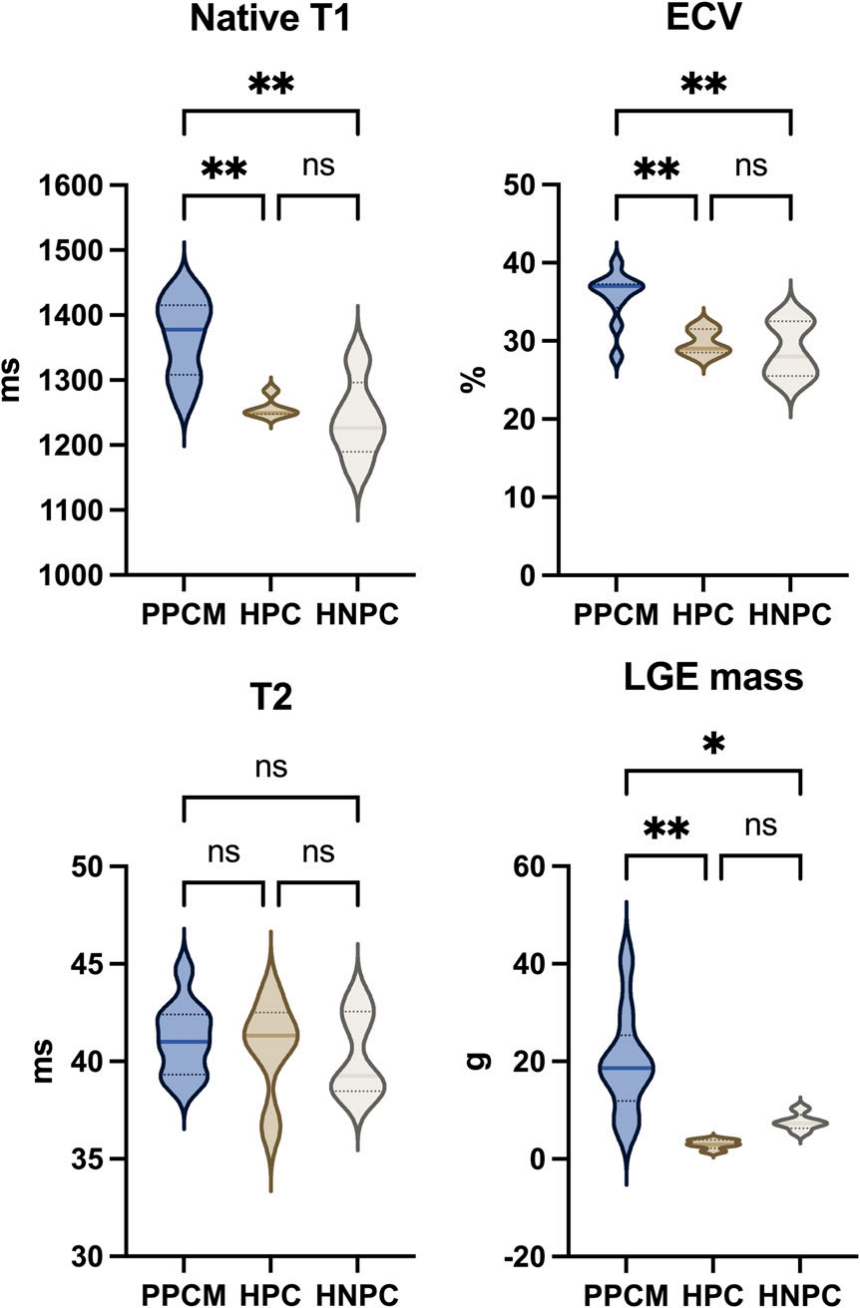
Results of cardiovascular magnetic resonance mapping studies as compared between peripartum cardiomyopathy (PPCM) patients, healthy postpartum controls (HPC) and healthy non‐postpartum controls (HNPC). ECV, extracellular volume fraction; LGE, late gadolinium enhancement. *p ≤ 0.05, **p ≤ 0.01.

Late gadolinium enhancement imaging was performed in all participants including controls, however there were significant artefacts in one PPCM participant that precluded further LGE analysis leaving nine usable LGE datasets from the PPCM participants. Importantly, LGE (as a sign of regional myocardial fibrosis) was only visible in participants with PPCM (present in 80% of the cohort). Correspondingly, ECV and native T1 time as markers of interstitial fibrosis were solely elevated in participants with PPCM compared to the control groups. T2 mapping, an index of myocardial oedema, showed no significant differences between groups (*Figure* *4*).

**Figure 5:**
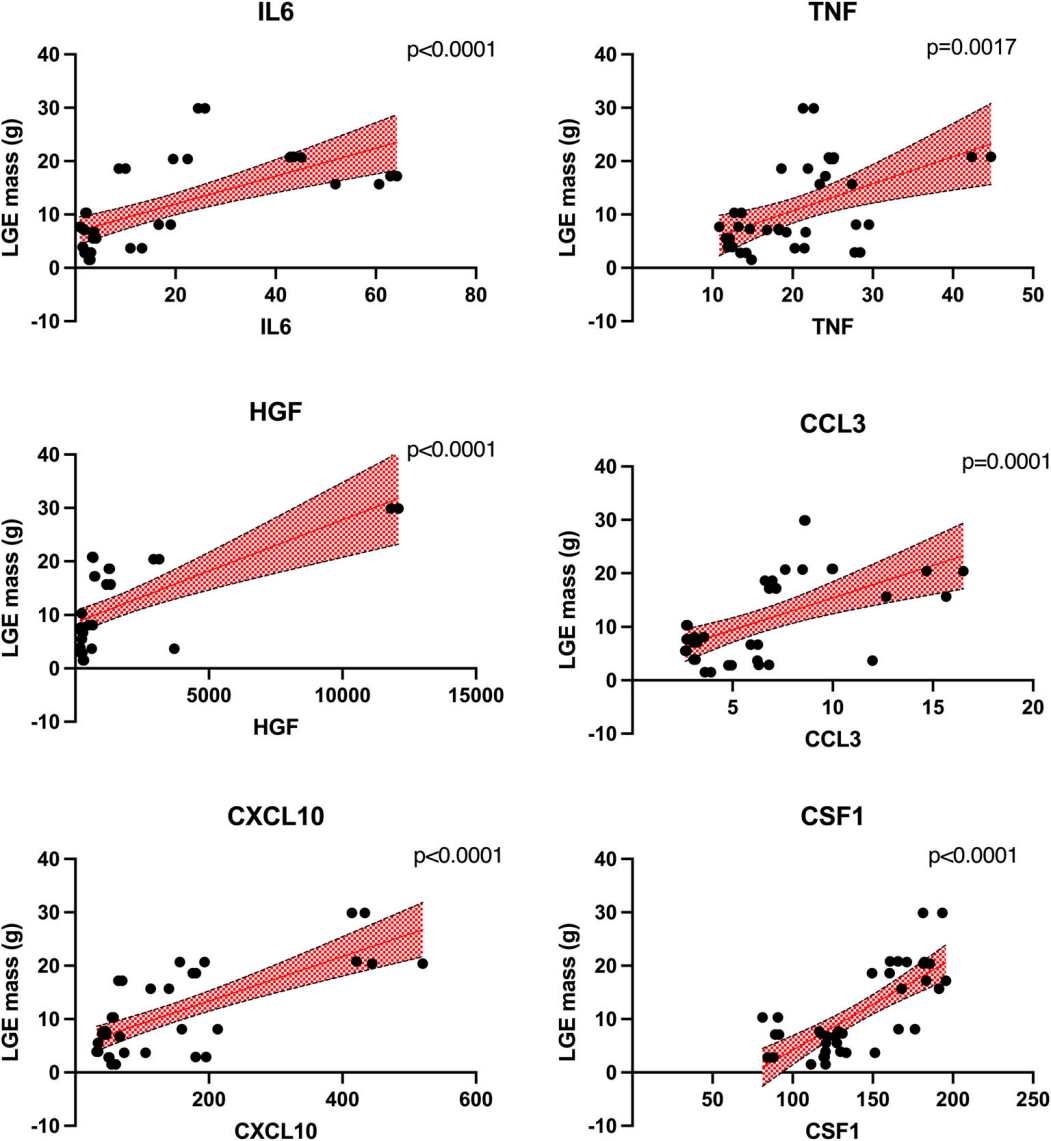
Correlations between late gadolinium enhancement (LGE) mass and circulatory inflammatory cytokines. CCL3, chemokine (C‐C motif) ligand 3; CSF1, colony stimulating factor‐1; CXCL10, chemokine (C‐X‐C motif) ligand 10; HGF, hepatocyte growth factor; IL6, interleukin‐6; TNF, tumor necrosis factor.

As shown in *Figure* *5*, LGE mass correlated moderately with IL‐6 (*r* = 0.43), CSF1 (*r* = 0.55), CXCL10 (*r* = 0.55), HGF (*r* = 0.41) (all *p* < 0.001), and weakly with CCL3 (*r* = 0.33, *p* = 0.0001), and TNF (*r* = 0.24, *p* = 0.0017).

### ^18^FDG‐PET‐CT

**Figure 6:**
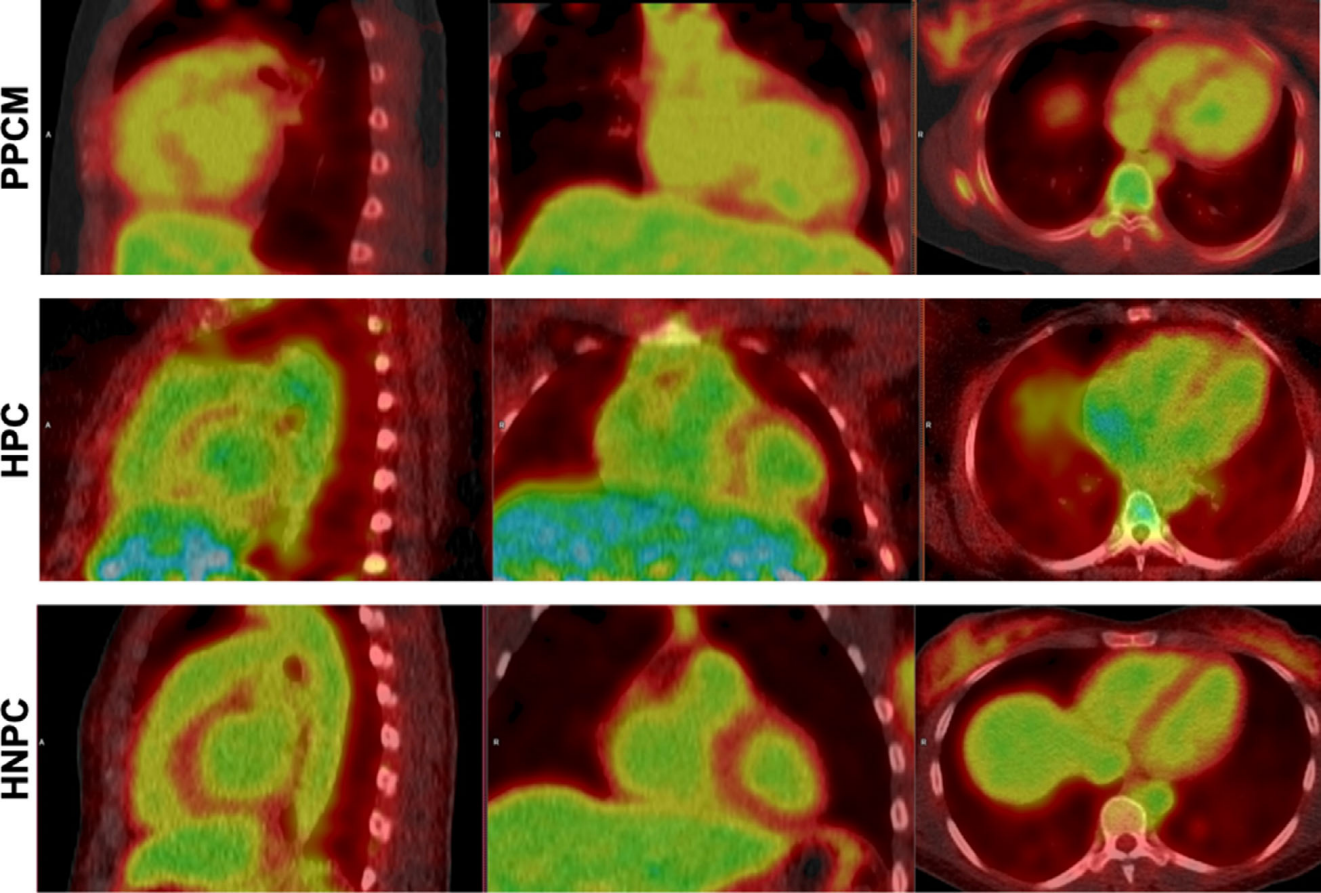
Representative cardiac ^18^FDG‐PET‐CT scan images of peripartum cardiomyopathy (PPCM) patients, healthy postpartum controls (HPC) and healthy non‐postpartum controls (HNPC) displaying a sagittal (first row), frontal (second row) and transverse (third row) plane.

**Figure 7:**
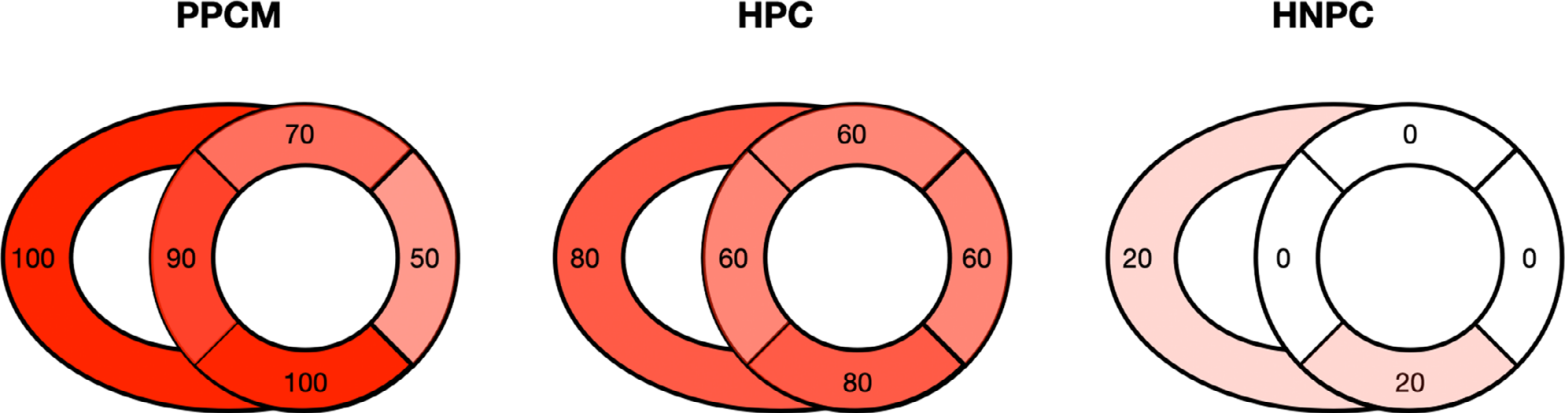
Distribution of ^18^FDG uptake in different cardiac regions as compared between peripartum cardiomyopathy (PPCM) patients, healthy postpartum controls (HPC) and healthy non‐postpartum controls (HNPC).

Blood glucose levels, FDG dose, and imaging delay for both whole‐body and cardiac image acquisition were comparable across the groups during the ^18^FDG‐PET‐CT scan (*Table* *3*). Patients with PPCM exhibited a significantly higher total cardiac ^18^FDG uptake compared to HNPC (1.7 [IQR 1.6–1.8] vs. 1.2 [IQR 1.2–1.3], *p* = 0.007). Total cardiac ^18^FDG uptake was also higher in HPC (2.0 [IQR 1.7–2.2]) than in HNPC (*p* = 0.016), however, there were no significant differences between cardiac ^18^FDG uptake in PPCM and HPC. Right ventricular uptake was significantly higher in PPCM than in HNPC (1.2 [IQR 0.8–1.3] vs. 0.4 [IQR 0.3–0.5], *p* = 0.049). An illustration of representative cardiac ^18^FDG‐PET‐CT images is provided in *Figure* *6*. A detailed description of the ^18^FDG uptake in different cardiac regions can be found in *Figure* *7*. On the whole‐body scans, TBR of the liver, breast and uterus showed no significant differences between groups. Spleen uptake was significantly higher in PPCM (1.9 [IQR 1.7–2.1]) compared to HNPC (1.4 [IQR 1.2–1.7], *p* = 0.037). However, spleen uptake showed no significant differences between PPCM and HPC.

**Table 3:** ^18^FDG‐PET‐CT imaging of patients with peripartum cardiomyopathy, healthy postpartum controls and healthy non‐postpartum controls

	Total (*n* = 20)	PPCM (*n* = 10)	HPC (*n* = 5)	HNPC (*n* = 5)	*p*‐value
Glucose (mmol/L)	4.9 (4.3–5.2)	4.3 (3.4–4.9)	5.2 (5.1–5.4)	4.9 (4.8–5.1)	0.12
^18^FDG dose (MBq)	204.0 (171.0–238.0)	213.0 (170.0–236.0)	243.0 (172.0–244.0)	181.0 (180.0–198.0)	0.34
Imaging delay whole body (min)	63.0 (60.0–67.0)	62.5 (60.0–67.0)	63.0 (60.0–65.0)	63.0 (60.0–79.0)	0.87
Imaging delay cardiac (min)	45.0 (45.0–50.5)	45.5 (45.0–49.0)	45.0 (45.0–45.0)	46.0 (45.0–66.0)	0.74
TBR					
Total cardiac	1.7 (1.3–1.9)	1.7 (1.6–1.8)	2.0 (1.7–2.2)	1.2 (1.2–1.3)	0.012
Septal wall	1.0 (0.9–1.2)	1.1 (1.0–1.2)	1.6 (1.0–1.9)	0.8 (0.8–0.9)	0.015
Anterior wall	0.8 (0.6–1.3)	0.7 (0.6–1.3)	1.4 (0.6–1.7)	0.7 (0.6–0.9)	0.59
Lateral wall	0.9 (0.6–1.4)	1.0 (0.8–1.3)	1.6 (0.9–1.7)	0.4 (0.4–0.6)	0.017
Inferior wall	1.2 (0.8–1.4)	1.2 (1.1–1.4)	1.5 (1.1–1.6)	0.7 (0.7–0.8)	0.006
Right ventricle	0.9 (0.5–1.2)	1.2 (0.8–1.3)	1.1 (0.9–1.3)	0.4 (0.3–0.5)	0.010
Spleen	1.7 (1.6–1.9)	1.9 (1.7–2.1)	1.7 (1.7–1.7)	1.4 (1.2–1.7)	0.074
Liver	2.2 (1.9–2.4)	2.3 (2.1–2.5)	2.2 (2.1–2.2)	1.9 (1.8–2.3)	0.39
Breast	1.3 (1.1–1.5)	1.4 (1.3–1.5)	1.5 (1.1–1.6)	1.1 (1.0–1.3)	0.55
Bone marrow	1.9 (1.7–2.2)	2.1 (1.8–2.5)	1.8 (1.7–1.8)	2.1 (1.6–2.1)	0.22
Uterus	1.9 (1.7–3.1)	2.0 (1.8–3.1)	2.1 (1.9–3.8)	1.8 (1.7–1.9)	0.33

^18^FDG, ^18^F‐fluorodeoxyglucose; CT, computed tomography; HNPC, healthy non‐postpartum controls; HPC, healthy postpartum controls; PET, positron emission tomography; PPCM, peripartum cardiomyopathy; TBR, target‐to‐background ratio.

## Discussion

This study shows that women with PPCM have extensive cardiac inflammation. Imaging revealed a correlation with an inflammatory proteomic profile, suggesting dysregulated innate immune activation and early diffuse cardiac fibrosis on CMR. This pattern contrasts with the immune activation seen in normal postpartum physiological adaptations. Additionally, whole‐body ^18^FDG‐PET‐CT showed splenic activation in PPCM and HPC indicative of strong circulatory immune activation in the postpartum period. In contrast, although healthy postpartum women showed increased cardiac ^18^FDG uptake, they had attenuated pro‐inflammatory immune activation on proteomic profiling, and CMR showed no signs of interstitial fibrosis. This highlights key differences between physiological postpartum remodelling and the pathological processes involved in PPCM (*Graphical Abstract*).

Patients with PPCM showed elevated leucocyte levels and increased NLR compared to controls. NLR has emerged as a marker of systemic inflammation and dysregulated leucocyte activation. In the BIOSTAT‐CHF patient cohort, NLR was significantly associated with the primary outcome of time to all‐cause mortality or hospitalization for HF irrespective of LV ejection fraction. Patients with an NLR in the highest tertile experienced poor outcomes significantly more frequently compared to those in the lowest tertile.^17^ NLR has also been suggested as a prognostic factor in predicting persistent LV dysfunction in patients with PPCM.^18^

Pro‐inflammatory cytokines such as IL‐6, TNF play a crucial role in perpetuating systemic inflammatory pathophysiology in HF as well as in the acute phase of PPCM.^9^, ^19^ Using inflammatory proteomic profiling, we confirmed upregulation of IL‐6 and TNF as well as additional inflammatory cytokines such as CXCL10, HGF, CSF1, CCL3 which were significantly upregulated in PPCM compared to healthy postpartum women. These have a discriminatory potential to differentiate postpartum immune activation from PPCM and may be crucially involved in the pathophysiology of PPCM. The pro‐inflammatory cytokines IL‐6 and TNF have been shown to exert negative inotropic effects on cardiomyocytes.^20^, ^21^ Moreover, TNF activates matrix metalloproteinases, which break down the extracellular collagen matrix, leading to LV dilatation.^22^ Previous studies have demonstrated that adding the anti‐inflammatory agent pentoxifylline, which inhibits the production of TNF and IL‐6, to standard HF therapy improved LV ejection fraction and symptoms in patients with ischaemic and dilated cardiomyopathy, as well as in those with PPCM.^14^, ^23^, ^24^, ^25^

Chemokines are a specialized family of cytokines that regulate biological processes such as chemotaxis, collagen turnover, angiogenesis, and apoptosis.^26^ This is important for the understanding of the pathophysiology of PPCM. Seminal experimental research in PPCM has shown that full‐length prolactin (23‐kDa) and interferon gamma (IFN‐γ), through Akt activation, promote the upregulation of chemokine (C‐C motif) ligand 2 (CCL2) in cardiomyocytes, which subsequently leads to local myocardial inflammation.^10^ Patients with PPCM showed increased levels of CCL2 and CCL3. Similarly, studies on patients with congestive HF have also reported increased levels of both CCL2 and CCL3.^27^

In PPCM, oxidative stress is thought to trigger cathepsin D to cleave 23‐kDa prolactin into a 16‐kDa fragment, which leads to endothelial dysfunction through the up‐regulation of miRNA‐146a. This process causes vasoconstriction, inflammation, and cardiomyocyte apoptosis, ultimately resulting in myocardial dysfunction and LV dilatation.^7^, ^28^ Furthermore, 16‐kDa prolactin promotes adhesion of inflammatory cells to the endothelium and stimulates the expression of IFN‐γ responsive genes, which then leads to a sustained inflammatory state.^9^, ^29^ Importantly, it has previously been shown that elevated IFN‐γ levels correlated with lower LV recovery in PPCM.^9^

Elevated serum levels of CXCL10 have previously been reported in patients with HF,^30^ which is consistent with the findings of this study. The chemokine CXCL10, also known as interferon gamma‐induced protein 10 (IP‐10), plays a crucial role in the inflammatory response. It is secreted by various cell types in response to IFN‐γ.^31^ HGF was found to be markedly up‐regulated in women with PPCM. Originally purified and cloned as a potent mitogen for hepatocytes, HGF exhibits mitogenic, anti‐apoptotic, pro‐angiogenic, and anti‐fibrotic effects across various cell types.^32^ It is notably elevated in incident HF and has been identified as an independent predictor of mortality in HF.^33^, ^34^ In addition, there is the down‐regulation of cytokines, such as IL‐4 and IL‐13, which have been shown to promote M2‐macrophage polarization. M2 macrophages regulate the resolution phase of inflammation and the repair of damaged tissues.^35^ The down‐regulation of these cytokines may therefore suggest a possible mechanism for the pathophysiological processes seen and adverse remodelling in PPCM, as opposed to the physiological response to inflammation in healthy postpartum women.

The spleen serves as the largest organ of the reticuloendothelial system and plays a major role in immunity.^36^ As an important reservoir for the deployment of leucocytes, it has a crucial role in immune responses.^37^ In acute myocardial infarction, it has been shown that the increased sympathetic nervous system activity promotes monocytes to mobilize progenitor cells from the bone marrow, which subsequently seed in the spleen and boost monocyte production.^38^ Spleen activation, indicated by increased ^18^FDG uptake, has been observed in humans following acute coronary syndromes. This activation correlates with elevated circulatory CRP and TNF levels, and the metabolic activity of the spleen independently predicted subsequent cardiovascular disease events.^39^ Moreover, circulatory CSF1 levels were significantly elevated in women with PPCM. CSF1 regulates the development of blood monocytes from haematopoietic stem cells, which may account for the increased splenic ^18^FDG activity observed in these patients.^40^

Both monocytes and macrophages play a central role in mediating tissue damage and myocardial fibrosis.^26^ HPC showed evidence of cardiac inflammation by means of ^18^FDG‐PET‐CT compared to HNPC. However, they did not show any evidence of diffuse myocardial fibrosis as assessed by LGE, T1 mapping, ECV and LGE mass. IL‐6 has the capability to recruit macrophages and directly stimulate fibroblasts. Prolonged activation of pro‐inflammatory macrophages leads to significant cardiac tissue remodelling by secreting matrix proteases and activating myofibroblasts.^41^, ^42^ In PPCM, the excess of pro‐inflammatory cytokines such as IL‐6 and TNF and downregulation of cytokines such as IL‐4 and IL‐13, which promote an M2‐macrophage polarization may be involved in the adverse LV remodelling seen on cardiac magnetic resonance imaging in patients with PPCM.^35^ Importantly, this cohort of patients with PPCM were recruited very early in their disease. It is striking that fibrosis was already present in the acute phase of the disease. Tackling the acute inflammatory and immune response leading to early onset fibrosis by targeted immunomodulatory therapy in addition to disease‐specific and guideline‐directed medical therapy (GDMT) might reduce the degree of irreversible LV remodelling in PPCM. Sodium–glucose co‐transporter 2 (SGLT2) inhibitors have been shown to have anti‐inflammatory effects by decreasing levels of IL‐6, CRP, TNF and monocyte chemoattractant protein‐1 in rodents.^43^ Moreover, the SGLT2 inhibitor empagliflozin significantly reduced ECV as assessed by CMR in a recent systematic review and meta‐analysis.^44^ Similarly, sacubitril/valsartan showed anti‐fibrotic effects in patients with HF and preserved ejection fraction.^45^ This highlights that the early onset fibrosis seen in PPCM may be attenuated by rapid initiation of GDMT especially with dugs documented to have anti‐fibrotic effects.

### Limitations

Considering that PPCM is a relatively rare condition, we acknowledge that the sample size in this single‐centre study is limited, which may affect the precision of our estimates. Nevertheless, this represents the first study of its kind and should be viewed as hypothesis‐generating, providing a foundation for future larger, multicentre investigations. A major limitation of the current study, however, is the lack of a comparator subgroup with HF unrelated to pregnancy, such as an age‐matched group with dilated cardiomyopathy. While our data demonstrate differential activation of circulatory biomarkers in PPCM patients compared to HPC, it is possible that some of these alterations, such as elevated leucocyte counts and pro‐inflammatory cytokines, may not be unique to PPCM but rather represent more general features of acute HF. Thus, without a HF control group, we cannot exclude the possibility that the biomarker profile observed in PPCM reflects a broader pathophysiological response common to other forms of HF with reduced ejection fraction. Future studies including such comparator groups will be essential to clarify which immune and inflammatory signatures are specific to PPCM. Whereas our study used circulatory inflammatory biomarkers and visualized myocardial inflammation using ^18^FDG‐PET‐CT, the inflammatory processes and pathways regulated within the myocardium need to be better defined on a cellular level. Future research should therefore investigate the underlying cardiac cellular composition in relation to inflammation and immune activation in PPCM.

## Conclusion

This study showed that women with PPCM have a dysregulated immune response, leading to early diffuse fibrosis and cardiac remodelling. This contrasts with the physiological changes observed in healthy postpartum women. The identification of novel biomarkers in these processes enhances our understanding of the pathophysiology of PPCM, which is not confined to the heart. This may, in future, pave the way to the development of new targeted therapies specific to PPCM.

## Supporting information


**Appendix S1.** Supporting Information.
