# Supplementary material for: Immune dysregulation as a key driver of peripartum cardiomyopathy – an exploratory advanced imaging and biomarker study
Source: Eur J Heart Fail. 2025 Jul 14;27(10):1868–80. doi: 10.1002/ejhf.3759 (PMC12575422; doi:10.1002/ejhf.3759)
Supplement: Supplementary file 1 — Appendix S1. Supporting Information. [file EJHF-27-1868-s001.docx]

# **Supplemental material:**

## **Table S1: Inclusion and exclusion criteria**

**PPCM patients**:

Inclusion criteria for **PPCM patients**:

1. Women within the first three post-partal months
2. Women with a diagnosis of PPCM
3. Women with an LVEF <45%
4. Absence of any previous heart disease
5. Age ≥ 18 years
6. Signed informed consent form

Exclusion criteria for **PPCM patients**:

1. Patient unable to give written informed consent
2. Previous or current diagnosis of any cardiovascular disease
3. Type I and II Diabetes mellitus
4. Any infectious diseases such as HIV or TB
5. Patients on steroid therapy
6. GFR < 45 mL/min/1,73 m²

**Healthy postpartal controls:**

Inclusion criteria for **healthy postpartal controls**:

1. Postpartal women up to 6 months after delivery
2. Age ≥ 30 years (refer to section 8.1. for justification in age cut-off)
3. Absence of any previous heart disease
4. Signed informed consent form

Exclusion criteria for **healthy postpartal controls**:

1. Mothers that are breastfeeding their baby
2. Type I and II Diabetes mellitus
3. Any infectious diseases such as HIV or TB
4. Steroid therapy
5. GFR < 45 mL/min/1,73 m²

**Healthy non-postpartal controls**

Inclusion criteria for **healthy non-postpartal controls**:

1. Women who have not been pregnant before or have not delivered within the past 12 months.
2. Age ≥ 30 and ≤ 40years (refer to section 8.1. for justification in age cut-off)
3. Absence of any cardiovascular disease.
4. Signed informed consent form.

Exclusion criteria for **healthy non-postpartal controls**:

1. Mothers who have given birth within the past 12 months
2. Type I and II Diabetes mellitus
3. Any infectious diseases such as HIV or TB
4. Steroid therapy
5. GFR < 45 mL/min/1,73 m²

## **Table S2: Complete biomarker list including UniProtKB**

| **Protein name** | **UniProtKB** |
| --- | --- |
| **Chemokines** | |
| Fms-related tyrosine kinase 3 ligand (FLT3LG) | P49771 |
| Granulocyte colony-stimulating factor (CSF3) | P09919 |
| Granulocyte-macrophage colony-stimulating factor (CSF2) | P04141 |
| Interferon gamma (IFNG) | P01579 |
| Interleukin-1 beta (IL1B) | P01584 |
| Interleukin-2 (IL2) | P60568 |
| Interleukin-4 (IL4) | P05112 |
| Interleukin-6 (IL6) | P05231 |
| Interleukin-7 (IL7) | P13232 |
| Interleukin-8 (CXCL8) | P10145 |
| Interleukin-10 (IL10) | P22301 |
| Interleukin-13 (IL13) | P35225 |
| Interleukin-15 (IL15) | P40933 |
| Interleukin-17A (IL17A) | Q16552 |
| Interleukin-17C (IL17C) | Q9P0M4 |
| Interleukin-17F (IL17F) | Q96PD4 |
| Interleukin-18 (IL18) | Q14116 |
| Interleukin-27 (IL27) | Q8NEV9, Q14213 |
| Interleukin-33 (IL33) | O95760 |
| Lymphotoxin-alpha (LTA) | P01374 |
| Macrophage colony-stimulating factor 1 (CSF1) | P09603 |
| Oncostatin-M (OSM) | P13725 |
| Tumor necrosis factor (TNF) | P01375 |
| Tumor necrosis factor ligand superfamily member 10 (TNFSF10) | P50591 |
| Thymic stromal lymphopoietin (TSLP) | Q969D9 |
| Tumor necrosis factor ligand superfamily member 12 (TNFSF12) | O43508 |
| **Chemokines** | |
| C-C motif chemokine 2 (CCL2) | P13500 |
| C-C motif chemokine 3 (CCL3) | P10147 |
| C-C motif chemokine 4 (CCL4) | P13236 |
| C-C motif chemokine 7 (CCL7) | P80098 |
| C-C motif chemokine 8 (CCL8) | P80075 |
| C-C motif chemokine 13 (CCL13) | Q99616 |
| C-C motif chemokine 19 (CCL19) | Q99731 |
| C-X-C motif chemokine 9 (CXCL9) | Q07325 |
| C-X-C motif chemokine 10 (CXCL10) | P02778 |
| C-X-C motif chemokine 11 (CXCL11) | O14625 |
| Eotaxin (CCL11) | P51671 |
| Stromal cell-derived factor 1 (CXCL12) | P48061 |
| **Other** | |
| Hepatocyte growth factor (HGF) | P14210 |
| Interstitial collagenase (MMP1) | P03956 |
| Macrophage metalloelastase (MMP12) | P39900 |
| Oxidized low-density lipoprotein receptor 1 (OLR1) | P78380 |
| Pro-epidermal growth factor (EGF) | P01133 |
| Protransforming growth factor alpha (TGFA) | P01135 |
| Vascular endothelial growth factor A (VEGFA) | P15692 |

#### **Table S3: Inflammatory biomarker profiling between PPCM, HPC and HNPC.**

|  | **Total** | **PPCM** | **HPC** | **HNPC** | **p-value** | **F-Test threshold** |
| --- | --- | --- | --- | --- | --- | --- |
| CCL11 | 71.0 (57.6-94.8) | 75.2 (57.9-107.6) | 64.3 (56.1-89.8) | 69.7 (60.4-83.3) | 0.79 | Non-significant |
| CCL13 | 62.9 (40.0-86.2) | 54.5 (38.0-80.0) | 91.4 (71.8-174.9) | 66.6 (20.7-92.7) | 0.035 | Non-significant |
| CCL19 | 109.6 (85.4-179.2) | 123.1 (106.1-195.4) | 98.2 (80.4-122.1) | 93.1 (78.2-99.4) | 0.023 | Non-significant |
| CCL2 | 240.2 (181.3-295.9) | 276.7 (240.2-345.1) | 248.4 (191.8-297.5) | 153.3 (132.8-172.2) | <0.001 | Significant |
| CCL3 | 5.4 (3.1-8.1) | 7.4 (6.1-10.0) | 4.9 (3.6-6.3) | 2.8 (2.7-3.1) | <0.001 | Significant |
| CCL4 | 60.4 (41.7-92.6) | 72.9 (55.9-100.8) | 65.1 (42.5-101.5) | 35.8 (30.6-51.6) | 0.001 | Significant |
| CCL7 | 1.2 (0.5-1.8) | 1.6 (0.9-1.9) | 0.7 (0.6-1.8) | 0.5 (0.4-0.7) | 0.13 | Non-significant |
| CCL8 | 32.4 (24.0-43.0) | 36.2 (25.0-42.0) | 66.7 (34.3-76.8) | 15.0 (13.0-24.6) | <0.001 | Significant |
| CSF1 | 132.2 (119.7-178.8) | 178.8 (163.3-183.4) | 120.2 (111.5-129.8) | 117.1 (90.8-127.6) | <0.001 | Significant |
| CSF2 | 0.1 (0.1-0.2) | 0.2 (0.1-0.2) | 0.2 (0.1-0.2) | 0.1 (0.1-0.1) | 0.004 | Significant |
| CSF3 | 118.3 (89.8-163.8) | 109.9 (91.5-173.0) | 118.3 (114.6-163.9) | 124.1 (54.2-128.7) | 0.48 | Non-significant |
| CXCL10 | 66.4 (48.0-180.4) | 167.7 (68.5-417.5) | 57.3 (49.8-105.2) | 42.8 (40.3-46.2) | <0.001 | Significant |
| CXCL11 | 105.5 (43.2-222.2) | 127.6 (117.7-248.0) | 95.3 (43.4-408.1) | 47.4 (41.1-75.0) | 0.021 | Non-significant |
| CXCL12 | 181.5 (155.9-211.6) | 188.0 (161.5-221.6) | 182.5 (165.6-198.8) | 159.4 (134.6-210.2) | 0.41 | Non-significant |
| CXCL8 | 5.0 (4.0-11.6) | 9.6 (7.1-13.9) | 4.3 (4.0-4.6) | 2.9 (2.7-4.5) | <0.001 | Significant |
| CXCL9 | 49.4 (38.4-85.8) | 78.2 (48.2-109.4) | 49.6 (41.7-88.9) | 36.8 (27.8-42.6) | 0.008 | Significant |
| EGF | 41.4 (23.4-89.7) | 43.6 (18.6-105.7) | 77.2 (39.9-177.5) | 33.5 (28.9-42.4) | 0.43 | Non-significant |
| FLT3LG | 105.7 (93.5-123.0) | 112.5 (95.4-119.3) | 132.1 (80.8-140.8) | 98.1 (95.5-101.7) | 0.18 | Non-significant |
| HGF | 413.7 (235.9-1210.4) | 951.0 (655.4-2826.7) | 253.5 (227.5-319.6) | 231.9 (211.3-243.5) | <0.001 | Significant |
| IFN‑γ | 0.2 (0.1-0.2) | 0.2 (0.1-0.4) | 0.2 (0.1-0.2) | 0.1 (0.1-0.1) | 0.008 | Significant |
| IL10 | 5.1 (3.7-9.4) | 7.6 (4.2-10.8) | 4.5 (3.2-6.0) | 4.8 (2.6-5.5) | 0.070 | Non-significant |
| IL13 | 0.5 (0.2-1.0) | 0.3 (0.2-0.6) | 1.0 (0.9-2.6) | 0.3 (0.2-0.5) | 0.002 | Significant |
| IL15 | 15.1 (12.5-21.5) | 19.3 (16.2-24.5) | 14.3 (14.0-15.4) | 12.2 (11.7-13.6) | <0.001 | Significant |
| L17A | 0.5 (0.4-1.0) | 0.6 (0.5-1.2) | 0.5 (0.2-0.6) | 0.5 (0.4-0.6) | 0.26 | Non-significant |
| IL17C | 28.9 (19.7-46.0) | 37.7 (23.6-90.4) | 25.7 (18.8-36.1) | 21.5 (12.2-29.9) | 0.032 | Significant |
| IL17F | 0.9 (0.5-1.7) | 0.5 (0.4-1.5) | 1.3 (1.0-1.7) | 1.1 (0.7-1.9) | 0.040 | Non-significant |
| IL18 | 349.1 (286.3-402.9) | 382.3 (302.6-467.8) | 304.3 (273.7-348.4) | 238.0 (190.8-360.1) | 0.009 | Significant |
| IL1B | 0.2 (0.1-0.3) | 0.2 (0.1-0.4) | 0.3 (0.2-0.3) | 0.1 (0.1-0.1) | 0.011 | Significant |
| IL2 | 0.0 (0.0-0.0) | 0.0 (0.0-0.0) | 0.0 (0.0-0.1) | 0.0 (0.0-0.0) | 0.012 | Non-significant |
| IL27 | 10.3 (7.9-14.4) | 13.0 (9.8-14.9) | 13.0 (8.6-14.8) | 7.2 (4.0-8.5) | <0.001 | Significant |
| IL33 | 0.2 (0.1-0.4) | 0.2 (0.1-0.4) | 0.3 (0.1-0.5) | 0.1 (0.1-0.2) | 0.12 | Non-significant |
| IL4 | 0.1 (0.0-0.1) | 0.1 (0.0-0.1) | 0.1 (0.1-0.1) | 0.0 (0.0-0.0) | 0.014 | Significant |
| IL6 | 6.4 (2.1-34.3) | 34.3 (17.8-56.3) | 2.7 (1.8-3.1) | 1.8 (1.4-2.2) | <0.001 | Significant |
| IL7 | 1.3 (0.8-2.8) | 1.5 (0.8-2.9) | 1.2 (0.9-4.0) | 1.2 (0.8-1.5) | 0.42 | Non-significant |
| LTA | 8.1 (6.6-11.0) | 6.7 (5.5-10.1) | 8.9 (7.7-9.7) | 11.8 (8.1-13.5) | 0.008 | Significant |
| MMP1 | 799.6 (444.3-2676.5) | 861.3 (718.1-6800.7) | 2588.1 (418.8-4525.0) | 614.0 (442.6-690.0) | 0.059 | Non-significant |
| MMP12 | 210.1 (164.1-316.2) | 316.2 (107.0-446.8) | 179.6 (169.1-265.9) | 204.8 (183.5-211.7) | 0.26 | Non-significant |
| OLR1 | 68.0 (42.6-116.7) | 81.6 (42.6-108.3) | 54.1 (43.8-516.1) | 58.9 (41.6-135.7) | 0.83 | Non-significant |
| OSM | 3.1 (1.6-7.8) | 4.2 (2.6-8.6) | 1.5 (0.9-11.2) | 1.7 (0.9-3.4) | 0.024 | Non-significant |
| TGFA | 7.3 (4.4-10.8) | 9.3 (7.6-13.0) | 6.6 (4.6-11.1) | 3.8 (3.2-4.2) | <0.001 | Significant |
| TNF | 19.7 (14.4-24.6) | 24.1 (21.5-26.3) | 14.9 (13.5-21.5) | 13.4 (12.1-16.8) | <0.001 | Significant |
| TNFSF10 | 343.2 (305.8-452.3) | 327.1 (303.5-355.7) | 403.6 (325.7-482.0) | 445.2 (318.9-473.6) | 0.12 | Non-significant |
| TNFSF12 | 421.4 (365.1-497.1) | 393.1 (290.9-471.6) | 451.4 (385.6-469.8) | 485.0 (378.4-512.7) | 0.29 | Non-significant |
| TSLP | 0.0 (0.0-0.2) | 0.1 (0.0-0.2) | 0.0 (0.0-0.1) | 0.0 (0.0-0.1) | 0.81 | Non-significant |
| VEGFA | 329.0 (213.7-518.1) | 473.1 (401.5-883.5) | 225.0 (197.2-321.8) | 208.8 (190.5-294.7) | <0.001 | Significant |

#### **Table S4: Description of significantly upregulated proteins between the groups.**

| **Protein** | **Uniprot Function Description** | **PPCM - HNPC** | **PPCM - HPC** | **HPC - HNPC** |
| --- | --- | --- | --- | --- |
| CCL3 | Monokine with inflammatory and chemokinetic properties. | Up | Up | Up |
| IL6 | Potent inducer of the acute phase response. | Up | Up |  |
| CSF1 | Plays an essential role in the regulation of survival, proliferation and differentiation of hematopoietic precursor cells, especially mononuclear phagocytes, such as macrophages and monocytes. Promotes the release of proinflammatory chemokines, and thereby plays an important role in innate immunity and in inflammatory processes. | Up | Up |  |
| CXCL10 | Pro-inflammatory cytokine that is involved in a wide variety of processes such as chemotaxis, differentiation, and activation of peripheral immune cells, regulation of cell growth, apoptosis and modulation of angiostatic effects. | Up | Up |  |
| TNF | Cytokine that binds to TNFRSF1A/TNFR1 and TNFRSF1B/TNFBR. It is mainly secreted by macrophages and can induce cell death of certain tumor cell lines. It is potent pyrogen causing fever by direct action or by stimulation of interleukin-1 secretion and is implicated in the induction of cachexia. Under certain conditions it can stimulate cell proliferation and induce cell differentiation. | Up | Up |  |
| HGF | Potent mitogen for mature parenchymal hepatocyte cells, seems to be a hepatotrophic factor, and acts as a growth factor for a broad spectrum of tissues and cell types. | Up | Up |  |
| CCL2 | Signals through binding and activation of CCR2 and induces a strong chemotactic response and mobilization of intracellular calcium ions. Exhibits a chemotactic activity for monocytes and basophils but not neutrophils or eosinophils. | Up |  | Up |
| TGFA | TGF alpha is a mitogenic polypeptide that is able to bind to the EGF receptor/EGFR and to act synergistically with TGF beta to promote anchorage-independent cell proliferation in soft agar. | Up |  | Up |
| IL27 | Interleukin-27 functions in innate immunity. IL-27 has pro- and anti-inflammatory properties, that can regulate T-helper cell development, suppress T-cell proliferation, stimulate cytotoxic T-cell activity, induce isotype switching in B-cells, and that has diverse effects on innate immune cells. | Up |  | Up |
| CCL4 | Monokine with inflammatory and chemokinetic properties. Binds to CCR5. | Up |  | Up |
| CSF2 | Cytokine that stimulates the growth and differentiation of hematopoietic precursor cells from various lineages, including granulocytes, macrophages, eosinophils and erythrocytes. | Up |  | Up |
| IL1β | Potent proinflammatory cytokine. Initially discovered as the major endogenous pyrogen, induces prostaglandin synthesis, neutrophil influx and activation, T-cell activation and cytokine production, B-cell activation and antibody production, and fibroblast proliferation and collagen production. | Up |  | Up |
| CCL8 | Chemotactic factor that attracts monocytes, lymphocytes, basophils and eosinophils. May play a role in inflammatory host responses. | Up | Down | Up |
| CXCL8 | IL-8 is a chemotactic factor that attracts neutrophils, basophils, and T-cells, but not monocytes. It is also involved in neutrophil activation. It is released from several cell types in response to an inflammatory stimulus. | Up |  |  |
| IL18 | A proinflammatory cytokine primarily involved in polarized T-helper 1 (Th1) cell and natural killer (NK) cell immune responses (Probable). Upon binding to IL18R1 and IL18RAP, forms a signaling ternary complex which activates NF-kappa-B, triggering synthesis of inflammatory mediator | Up |  |  |
| IL15 | Cytokine that stimulates the proliferation of T-lymphocytes. | Up |  |  |
| VEGFA | Growth factor active in angiogenesis, vasculogenesis and endothelial cell growth. Induces endothelial cell proliferation, promotes cell migration, inhibits apoptosis and induces permeabilization of blood vessels | Up |  |  |
| IFN‑γ | IFN-gamma has important immunoregulatory functions. It is a potent activator of macrophages. | Up |  |  |
| CXCL9 | Cytokine that affects the growth, movement, or activation state of cells that participate in immune and inflammatory response. Chemotactic for activated T-cells. Binds to CXCR3. | Up |  |  |
| IL17C | Cytokine that plays a crucial role in innate immunity of the epithelium, including to intestinal bacterial pathogens, in an autocrine manner. Stimulates the production of antibacterial peptides and proinflammatory molecules for host defense by signaling through the NF-kappa-B and MAPK pathways. | Up |  |  |
| IL18 | A proinflammatory cytokine primarily involved in polarized T-helper 1 (Th1) cell and natural killer (NK) cell immune responses (Probable). Upon binding to IL18R1 and IL18RAP, forms a signaling ternary complex which activates NF-kappa-B, triggering synthesis of inflammatory mediators. | Up |  |  |
| IL4 | Participates in at least several B-cell activation processes as well as of other cell types. It induces the expression of class II MHC molecules on resting B-cells. |  | Down | Up |
| LTA | Lymphotoxin is produced by lymphocytes and is cytotoxic for a wide range of tumor cells in vitro and in vivo. | Down |  |  |
| IL13 | Cytokine that Inhibits inflammatory cytokine production. May be critical in regulating inflammatory and immune responses. |  | Down |  |
